# Per- and Polyfluoroalkyl Substances in Food Packaging: Migration, Toxicity, and Management Strategies

**DOI:** 10.1021/acs.est.3c03702

**Published:** 2024-03-19

**Authors:** Drake W. Phelps, Lindsey V. Parkinson, Justin M. Boucher, Jane Muncke, Birgit Geueke

**Affiliations:** † Independent Consultant, Raleigh, North Carolina 27617, United States; ‡ Food Packaging Forum Foundation, 8045 Zürich, Switzerland

**Keywords:** Per- and polyfluoroalkyl substances, PFAS, food packaging, food contact chemicals

## Abstract

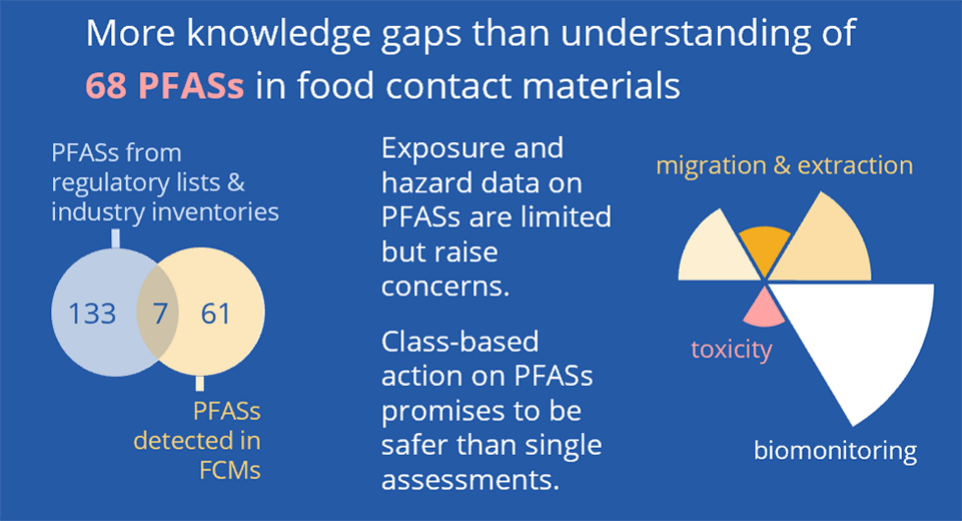

PFASs are linked to serious health and environmental concerns. Among their widespread applications, PFASs are known to be used in food packaging and directly contribute to human exposure. However, information about PFASs in food packaging is scattered. Therefore, we systematically map the evidence on PFASs detected in migrates and extracts of food contact materials and provide an overview of available hazard and biomonitoring data. Based on the FCCmigex database, 68 PFASs have been identified in various food contact materials, including paper, plastic, and coated metal, by targeted and untargeted analyses. 87% of these PFASs belong to the perfluorocarboxylic acids and fluorotelomer-based compounds. Trends in chain length demonstrate that long-chain perfluoroalkyl acids continue to be found, despite years of global efforts to reduce the use of these substances. We utilized ToxPi to illustrate that hazard data are available for only 57% of the PFASs that have been detected in food packaging. For those PFASs for which toxicity testing has been performed, many adverse outcomes have been reported. The data and knowledge gaps presented here support international proposals to restrict PFASs as a group, including their use in food contact materials, to protect human and environmental health.

## Abstract 2

PFAS are hazardous chemicals in food contact materials. Managing these compounds as a class is necessary to protect human health and the environment.

## Introduction

Per- and polyfluoroalkyl substances (PFASs) are a highly persistent class of chemicals of increasing concern that are accumulating in the environment, in humans, and are on track to exceed the planetary boundary for chemical pollution.^1^ More than 12,000 different PFASs are known to exist,^2^ and research has shown that PFASs are used globally in many consumer products and industrial processes.^3^ There is a growing discussion about how to effectively manage this large group of substances, including a recent restriction proposal to ban most PFASs in the European Union,^4^ the newly proposed maximum levels for six PFASs in drinking water in the US,^5^ and the listing of several PFASs as persistent organic pollutants by the Stockholm convention.^6^

PFASs are known to be found in food packaging and other food contact articles (FCAs) used in the production, processing, transport, handling, and storage of foods.^7^ Like many other food contact chemicals (FCCs), PFASs have been shown to migrate from different food contact materials (FCMs), such as paper and board and plastics, into food, allowing for exposure of the general public.^8−17^ Many current legal frameworks covering FCMs require the risk assessment of individual chemicals present in FCMs;^18^ however, to date there is no overview of the range of PFASs actually used in FCMs and whether they have been assessed for their risks at all. Furthermore, the presence of PFASs in FCMs raises particular concerns with regards to their life cycle, as the production of PFASs and PFAS-containing materials causes occupational exposure^19,20^ and their disposal leads to contamination of groundwater and drinking water via landfill leachate.^21,22^ Disposal of FCMs containing PFASs via recycling and/or incineration also may lead to environmental contamination and human exposure.^23^ Therefore, the question arises how regulators will address PFASs overall and in FCMs in particular in the future, especially in view of gaining regulatory attention, as illustrated by the European restriction proposal on PFASs,^4^ the current revision of the FCM legislation in the EU,^24^ and further global action.^25^

We have previously published systematic reviews detailing FCCs that have been identified in a wide range of FCAs and FCMs.^17,26^ The results of these reviews are publicly available data sets, the Food contact chemicals database (FCCdb) and the Database of migrating and extractable food contact chemicals (FCCmigex), that can inform the general public, fellow scientists, food packaging manufacturers, and policymakers. To date, we have identified more than 14,000 FCCs that have been reported by manufacturers and regulators and/or identified through literature searches of experimental migration and/or extraction studies. These chemicals may be present as intentionally or nonintentionally added substances in different types of FCAs. Several chemical groups with known hazard properties have been frequently identified in these data sets, including phthalates, bisphenols, heavy metals, and primary aromatic amines. For many other FCCs, hazard data are scarce. PFASs were also identified in these sources, but data availability strongly depends on the individual PFAS.

The Organization for Economic Co-operation and Development (OECD) defines PFASs as a class of “fluorinated chemicals that contain at least one fully fluorinated methyl or methylene carbon atom”.^27^ These moieties create hydrophobic and oleophobic barriers and are highly resistant to chemical and thermal degradation. PFASs have been in large-scale production since the 1950s to make a variety of products, including FCMs, water- and stain-resistant textiles, and fire-fighting foams.^3^ In paper- and plant-fiber-based FCMs, PFASs are used as sizing agents and chemical barriers against moisture and grease. For the production of plastic FCMs, PFASs are used as extrusion agents and mold release agents, and they are unintentionally formed during direct fluorination of polymers.^12^ Furthermore, they are, for example, applied in printing inks, filtering agents, and nonstick coatings.^3,4,28^ First migration studies on PFAS from FCMs were published in 2005,^29^ and migration of PFASs from paper and board food packaging was recently reviewed by Lerch et al.,^16^ concluding that these sources considerably contribute to dietary exposure.

Due to their unique chemistry, PFASs have enabled convenience in many parts of modern life. However, their molecular properties, have also granted them hazardous properties, including persistence, raising alarms due to their ubiquitous presence as contaminants in food, drinking water, and the environment. In the decades following their introduction, PFASs such as perfluorooctanoic acid (PFOA) and perfluorooctanesulfonic acid (PFOS) were identified in human serum samples.^30^ Today, various PFASs have been identified in sera from humans and wildlife globally.^31−33^ Exposure to some PFASs has been linked to a wide range of adverse health outcomes such as cancer, thyroid disease, decreased response to vaccination, and high cholesterol.^34^

Given the enormity of this class of compounds, however, exposure and hazard data are still extremely limited for most PFASs. In fact, most of what is known about the toxicity of PFASs has come from studies of some legacy PFASs that have, for example, been phased out of production in the United States since the early 2000s. Since then, other PFASs have entered the market to replace their legacy counterparts with little to no publicly available data on their hazards or risks to human and environmental health. To get an overview of the uses of PFASs in the sensitive application of FCMs, we mapped all known PFASs detected in FCMs using the publicly available FCCmigex database, reviewed available information on their hazards based on official classifications and hazard databases, and combined that information to review risk management approaches being proposed by governments and industry stakeholders. Ultimately, we would like to use this review to inform a class-based restriction of PFASs by focusing on their application in FCMs and the possible implications for human health and the environment.

## Methods

### Information Sources on FCCs and PFASs

The data on the migration and extraction of PFASs from food packaging and other FCAs were retrieved from a systematic evidence map on FCCs that makes the results accessible via an interactive tool, the FCCmigex dashboard.^17,35^ After the latest update in April 2023, the FCCmigex includes 24,848 database entries and 4,262 FCCs. These data are based on 1,312 publicly available studies and reports describing FCA migration and extraction experiments through October 2022 that were systematically mapped according to a previously published methodology.^17^ Each FCCmigex database entry provides information about a single FCC that was measured in extracts or migrates of an FCM, and it is always linked to the study from which it was generated. A database entry also contains precoded information about the type of FCM from which the FCC originates (e.g., plastics, paper and board, coatings), whether the investigated FCA was a single-use or repeat-use item, which experimental setup was used (migration into food or food simulant or extraction), and whether the FCC was detected or not. Depending on the experimental setup, analytical approach, and number of samples, between one and several hundred database entries per study were generated.

Here, FCCmigex data were filtered to identify only those compounds that have been detected in extracts or migrates of FCAs and FCMs. These FCCs were then cross-referenced, via Chemical Abstracts Service (CAS) Registry number, to the US Environmental Protection Agency (USEPA) PFAS Master List^2^ (last accessed 23 November 2022) to identify PFASs among the FCCs. This list from the USEPA includes more than 12,000 PFASs that have been consolidated and curated in an attempt to identify the known universe of PFASs. The PFASs in FCCmigex were then grouped by combining ionic, salt, and/or acid forms for corresponding PFASs (e.g., perfluorobutanoic acid and its salts were counted as one chemical), as detailed in Table S1.

We also compared the PFASs that were detected in migrates and extracts to the FCCdb, a comprehensive data set of over 12,000 FCCs from 67 publicly available global regulatory lists and industry inventories.^26^ The FCCdb database maps each chemical to its potential uses in 17 different FCM types.

### Mapping PFASs

In order to rank and compare these PFASs based on their presence in FCCmigex, toxicity data availability, and exposure data availability, we used the Toxicological Prioritization Index (ToxPi; v2.3).^36,37^ Each metric, referred to as a “slice,” in the model provided to ToxPi is given an individual slice score that ranges from 0 to 1.0 for each chemical. The slice score is calculated by normalizing all the metric’s data to the range 0 to 1.0 on a linear scale. Therefore, a slice score of 0 indicates that the chemical is ranked last for that metric relative to the other chemicals in the comparison. As described in the initial ToxPi publication,^36^ when visualizing the ToxPi analysis, the distance from the center for each slice is proportional to the respective slice score.

After slice scores are calculated, ToxPi then uses individual slice scores and integrates those scores into an overall ToxPi score for each chemical. The ToxPi score is a dimensionless measure that is the sum of the slice scores for an individual chemical normalized across all chemicals in the analysis from 0 to 1.0. In our analysis, the slices were weighted equally, resulting in the same width. A theoretical ToxPi score of 1.0 means that the chemical is ranked with the highest slice scores across all metrics; i.e., it has the highest values across all metrics. Conversely, a ToxPi score of 0 means that this chemical is ranked last because it has the lowest slice scores across all metrics; i.e., it has the lowest values across all metrics.

The overall ToxPi model and its slices were defined as the following: (1) the number of FCCmigex entries from extraction experiments, (2) the number of FCCmigex entries from migration into food simulant experiments, (3) the number of FCCmigex entries from migration into food experiments, (4) the number of ToxValDB/Hazard entries on USEPA’s CompTox Dashboard, (5) the number of ToxCast assays that have been performed according to USEPA’s CompTox Dashboard,^38,39^ and (6) the number, out of 6, of human biomonitoring studies in which the compound was detected in humans according to four different human biomonitoring programs (HBM4EU [Europe], NHANES [US], CHMS [Canada], Biomonitoring California [California, US]) and two metabolome/exposome databases.^40^

Presented data for trends over time were visualized using GraphPad Prism (v7.0e; GraphPad Software, San Diego, California, USA).

### Hazard Information

For PFASs that were identified in the FCCmigex and the FCCdb, we compiled additional information about their hazard properties according to the criteria within the European Chemicals Strategy for Sustainability.^41^ In brief, we recorded whether they are classified as carcinogenic, mutagenic, or toxic to reproduction (CMR) and have specific organ toxicity (STOT), endocrine disrupting properties, persistence and bioaccumulation-related hazards (PBT, vPvB), and/or persistent and mobile properties (PMT/vPvT). The consulted hazard data sources are listed in the Supporting Information, and the applied methodology was further detailed by Zimmermann et al.^42^ and Geueke et al.^43^

## Results and Discussion

### PFASs Detected in FCMs

Based on 47 studies, we have identified 552 entries for PFASs detected in migrates and/or extracts of FCMs from the FCCmigex database, consisting of 68 different PFASs found in paper/board, coated metal, and/or plastic FCMs. All PFASs with associated CAS numbers and the list of studies and reports can be found in Tables S1 and S2, respectively. Additional PFASs were identified in the literature but were not included in our analyses due to a low level of confidence during chemical identification^44^ or the lack of available CAS numbers reported by the authors.^8,9,45−51^ CAS numbers for these compounds were also not readily obtainable through searching the reported name via PubChem or the USEPA CompTox Dashboard. Due to the high number of synonyms for PFASs, we strongly encourage authors to always publish CAS numbers or other identifiers, such as USEPA’s Distributed Structure-Searchable Toxicity Identifier (DTXSID) or PubChem Identifier (PubChem CID), for reported compounds and support the registration of novel substances so that they may be appropriately identified.

**Figure 1:**
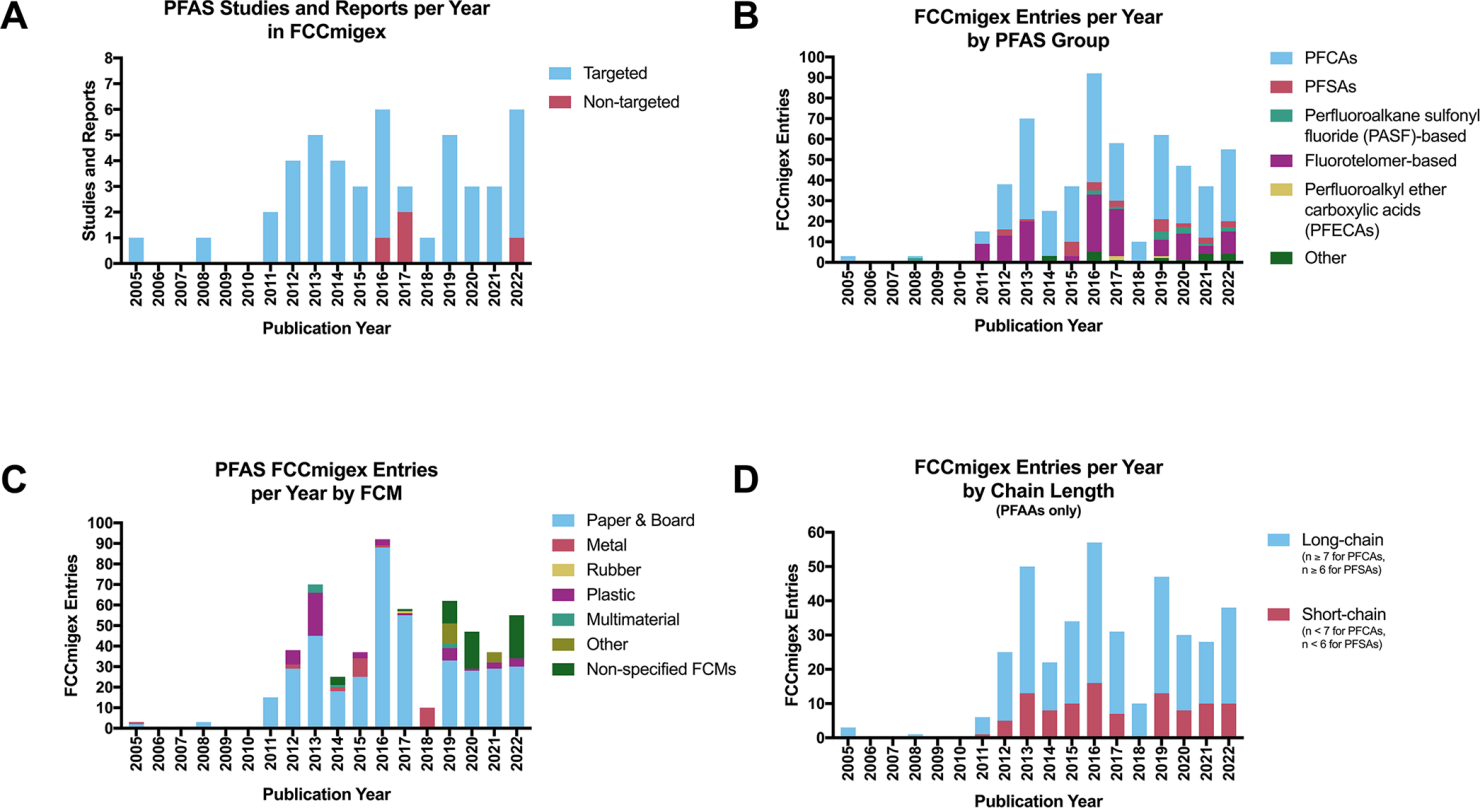
Evidence of PFASs in FCMs. (A) PFASs mostly found in targeted analyses: Studies and reports showing PFASs in FCMs were analyzed according to publication year and whether they used targeted or nontargeted approaches. (B) PFCAs and fluorotelomer-based PFASs dominate FCCmigex entries: PFASs that were detected in FCMs were categorized based on structures described in Wang et al.^56^ (PFCAs: perfluorocarboxylic acids, PFSAs: perfluorosulfonic acids, PASF: perfluoroalkane sulfonyl fluoride, PFECAs: perfluoroalkyl ether carboxylic acids). (C) Paper and board FCMs are where PFASs are most commonly detected: FCCmigex entries for PFASs were graphed based on the type of FCM where the PFAS was detected. (D) Long-chain PFAAs in FCCmigex are still regularly detected in FCMs: Given that PFAAs are a large portion of FCCmigex entries, the entries for PFAAs were categorized based on their chain length.^55^ Long-chain PFCAs and PFSAs carry an alkyl chain with at least seven and six carbon atoms, respectively. Short-chain PFCAs and PFSAs have an alkyl chain with not more than six and five carbon atoms, respectively.

Next, we sought to identify how experimental designs for PFAS-related studies and reports in FCCmigex have changed over time (Figure 1a). 43 of the 47 studies applied targeted analyses for the identification of PFASs, while four studies utilized nontargeted analyses. Given that thousands of PFASs are known to exist, targeted analyses have the potential to introduce the risk of bias by overlooking related compounds that are present in the sample, as shown by Schultes et al.^51^ In the future, novel developments in nontargeted screening may lead to additional PFASs being identified and quantified in FCMs.^52−54^ However, as these methods are time- and resource-intensive, they are likely not suitable for routine screening and managing the risks of PFASs in FCMs.

We then grouped the 68 detected PFASs by chemical structure according to Wang et al.^56^ into perfluorocarboxylic acids (PFCAs), perfluorosulfonic acids (PFSAs), perfluoroalkane sulfonyl fluoride (PASF)-based PFASs, fluorotelomer-based PFASs, perfluoroalkyl ether carboxylic acids (PFECAs), and other PFASs. To determine which subgroups of PFASs have been detected in migrates and extracts of FCMs, we analyzed the number of FCCmigex entries per group and year (Figure 1b). PFCAs and fluorotelomer-based compounds make up most of these entries, with 350 (63.4%) and 132 (23.9%), respectively. Given that fluorotelomer- and PASF-based compounds can degrade to PFAAs,^57−62^ they may indirectly contribute to the detected levels of PFCAs in FCMs. Such knowledge may be used to trace the origin of PFCAs in FCMs. However, it remains difficult to predict the precursor molecules because PFCAs present in FCMs could be intentionally added substances, degradation products of fluorotelomer-based PFASs or other subclasses of PFASs, or caused by the fluorination of plastics.

To determine what types of FCMs contain PFASs, we next grouped FCCmigex PFAS entries based on FCM. Paper and board were the most common FCMs where PFASs have been studied (Figure 1c), accounting for 72.5% of PFAS entries in FCCmigex. This finding can be explained by the common use of PFASs in paper and board food packaging that increases the grease and water barrier performance.^28^ However, it could also reflect the high interest in paper and board, as 68% of the studies analyzed this FCM. Recently, Lerch et al. published a comprehensive review about the migration of PFASs from paper and board FCMs, in which the analytical approaches and factors influencing migration behavior were investigated in detail.^16^ Based on these data, which were collected from migration studies published between 2008 and 2013, a risk estimation showed that the dietary exposure of the sum of all PFASs in FCMs, which included PFCAs, PFCAs, and fluorotelomer-based PFASs, exceeded the tolerable weekly intake of 4.4 ng per kg body weight, which was established by EFSA.^16,63^ Compared to paper and board food packaging, much less evidence exists for other origins of PFASs in FCMs. According to FCCmigex, plastic and coated metal FCMs also contain PFASs, which compromised 8.9% and 4.5% of the PFAS entries in FCCmigex, respectively. Treatment of plastics with fluorine gas has recently come under scrutiny, as this process creates PFASs^64^ that can migrate out of the plastic.^12^ PFASs were also found in FCMs made out of rubber, multimaterials, and nonspecified FCMs.

Next, upon querying the database, we found that long-chain perfluoroalkyl acids (PFAAs), including PFOA and PFOS, were most frequently detected in FCMs, but short-chain PFAAs also appeared regularly, beginning in 2011 (Figure 1d). In 2006, the PFOA Stewardship Program envisioned to strongly reduce and eventually phase out the use of long-chain PFAAs in the United States by 2010 and 2015, respectively.^65^ Already in 2014, scientists raised concerns about the industry’s transition from using long-chain PFAS to short-chain fluorinated alternatives in the Helsingør Statement, because short-chain PFASs are equally persistent, may be used in higher concentrations due to lower performance, have less toxicity data, and contribute to the overall exposure to a mixture of many different PFASs.^66^ Our data illustrate that long-chain PFAAs are now found alongside short-chain PFAAs, rather than being completely replaced by them. However, a more recent study that was published after the cutoff data for this review reported the detection of six-perfluorocarbon homologues with the highest abundance and relates this result to the industrial transition to short-chain PFASs.^59^ In the future, analyzing the levels of PFASs in food packaging could provide more insight into specific use patterns over time. Additionally, it could help to answer the question of whether long-chain PFAAs are still intentionally present substances, unintentionally formed degradation products of, e.g., fluorotelomers or fluorinated polymers, or created as byproducts in the manufacture of other PFASs used for food packaging.

Three PFASs have been added to the FCCmigex database from recent scientific studies, including the following: *N*-methylperfluorooctanesulfonamide (NMePFOSA),^67^ 1,2,3,4,5,5,6,6-octafluorobicyclo[2.2.2]oct-2-ene, and 2H-perfluoro-2-propanol.^68^ NMePFOSA was detected in a paper-based tea cup sampled from a coffee shop in Taiwan, and the latter two compounds were identified in the extracts of virgin PET samples intended for food contact through a nontargeted analysis, thus highlighting the utility of these methods to identify previously unobserved compounds in FCMs. Another compound of interest in our analysis is bisphenol AF (BPAF). BPAF was one of several bisphenol A (BPA) analogues phased into the market after rising concerns over the toxicity of BPA, but it has been reported to be as toxic, if not more toxic, than BPA, depending on the end point of interest.^69−78^ Of the BPA analogues, only BPAF is classified as a PFAS due to its two trifluoromethyl (−CF_3_) groups.^79^ BPAF is used in the production of fluoroelastomers, specialty rubber, and other polymers,^80^ and it has been observed almost exclusively in metal- and plastic-derived packaging, according to FCCmigex. Studies on BPAF in paper and board are limited, with only one of these four studies detecting BPAF in paper and board FCAs^81^ and three studies targeting but not detecting BPAF in these materials.^82−84^

### PFASs Reported for Intentional Use and Present in FCMs

The FCCdb, the database of intentionally added FCCs,^26^ contains 140 PFASs, which are detailed in the Supporting Information. Only seven of the 140 PFASs were also among the 68 PFASs found in FCCmigex (Table 1). This suggests, however, that 61 of the 68 PFASs in FCCmigex are unintentionally present in FCMs and that 133 PFASs in FCCdb do not have evidence of their presence in FCMs based on migration and extraction studies. Three of the seven compounds found in both databases–PFOA, GenX, and PFBS–have hazard properties targeted for restriction under the European Chemicals Strategy for Sustainability.^41,42^ BPAF meets the definition of an environmental endocrine disruptor according to recent information related to the restriction of bisphenols.^79^ Two other PFASs in Table 1 have very limited hazard data, but both of them can be degraded to PFASs that are of concern: bis(*N*-ethyl-2-perfluorooctylsulfonaminoethyl)phosphate is a precursor of PFOS, which is toxic to reproduction and a suspected carcinogen, and *N*-methylperfluorobutane sulfonamidoethanol can be degraded to PFBS (see Table 1). ADONA was characterized as very persistent and mobile, with concern for being bioaccumulative.^85^ Human health and endocrine disrupting properties were not evaluated in this assessment of ADONA;^85^ however, the toxicity of ADONA was recently reviewed and compared to other PFECAs.^86^

**Table 1:** PFASs Listed in the FCCdb and FCCmigex Databases, Their Hazard Properties, Potential Uses, and Evidence for the Presence in Different Types of FCMs^a^

**Food contact chemical**		**Hazard properties**		**Availability of toxicity data**		**Potential use and presence in FCMs**	
**Name**	**CAS number(s)**	**Food contact chemical ofconcern**(42)	**Other/not yet confirmed hazard properties ofconcern**(87)	**Number of ToxValDB/CompTox Hazard Entries**	**Number of ToxCast assays tested**	**FCC listed in FCM-specific source in theFCCdb**(26)	**FCC detected in migrate/extract of FCM (number of FCCmigex databaseentries)**(17)
Perfluorooctanoic acid (PFOA) and its salts	335-67-1 (acid); 3825-26-1 (ammonium salt); 335-95-5 (sodium salt); 2395-00-8 (potassium salt)	•CMR	•SVHC (PBT)	290	1396	•Plastics	•Paper and board (37)
						•Coatings	•Plastics (8)
		•STOT RE				•Printing inks	•Metals (5)
							•Multimaterials (1)
		•POP					•Rubber (1)
							•Other FCMs (2)
							•Nonspecified FCMs (6)
Perfluoro[2-(n-propoxy)propanoic acid] (GenX) and its salts	13252-13-6 (acid); 62037-80-3 (ammonium salt)	•vPvM and PMT	•SVHC (probable serious effects to human health and the environment)	7	506	•Plastics	•Paper and board (1)
Perfluorobutanesulfonic acid (PFBS) and its salts	375-73-5 (acid), 29420-49-3 (potassium salt), 45187-15-3 (sulfonate)	•No priority hazards reported	•SVHC (PBT; probable serious effects to human health and the environment)	138	1391	•Silicones	•Paper and board (4)
						•Printing inks	
ADONA	958445-44-8	•No priority hazards reported	•No	0	0	•Plastics	•Paper and board (1)
						•Coatings	
BPAF	1478-61-1	•No priority hazards reported	•Environmental endocrine disruptor^79^	28	1189	•Rubber	•Plastics (9)
							•Coated metals (3)
							•Paper and board (1)
							•Multimaterials (1)
Bis(*N*-ethyl-2-perfluorooctylsulfonaminoethyl)phosphate	30381-98-7 (ammonium salt), 23282-60-2 (sodium salt)	•No priority hazards reported	•No	3	0	•Paper and board	•Paper and board (3)
*N*-Methylperfluorobutane sulfonamidoethanol	34454-97-2	•No priority hazards reported	•No	7	0	•Printing inks	•Paper and board (1)

a Abbreviations: CMR = carcinogenic, mutagenic, or toxic to reproduction, STOT RE = specific organ toxicity, repeated exposure, POP = persistent organic pollutant, vPvM = very persistent, very mobile, PMT = persistent, mobile, and toxic, SVHC = Substance of Very High Concern, PBT = persistent, bioaccumulative, and toxic

The FCCdb inventories which FCCs are potentially used in each material type based on the information from the included regulatory and industry lists. Accordingly, GenX and ADONA are intentionally used as processing aids in the production of fluoropolymer-based plastics and coatings. This would suggest their presence in, e.g., fluoropolymer-coated pans, but they have only been detected in paper and board FCMs. Similarly, BPAF is indicated for use in rubber but was mainly detected in plastics and coated metals. PFBS and *N*-methylperfluorobutane sulfonamidoethanol are listed for use in printing inks and were found in paper and board FCMs, which can be explained by the frequent use of printed paper-based FCMs that often have low migration barriers. These data show that PFASs with known hazard properties of concern continue to be found on regulatory lists and in FCM inventories. Additionally, specific PFASs have been detected in migrates or extracts of FCM types where they would not have been expected based on the information in the FCCdb. By analysis of the information on use and migration of this small subset of PFASs, the difficulties in managing individual PFASs and tracing back their origins become apparent.

### Mapping Toxicity Information on PFASs Detected in FCMs

**Figure 2:**
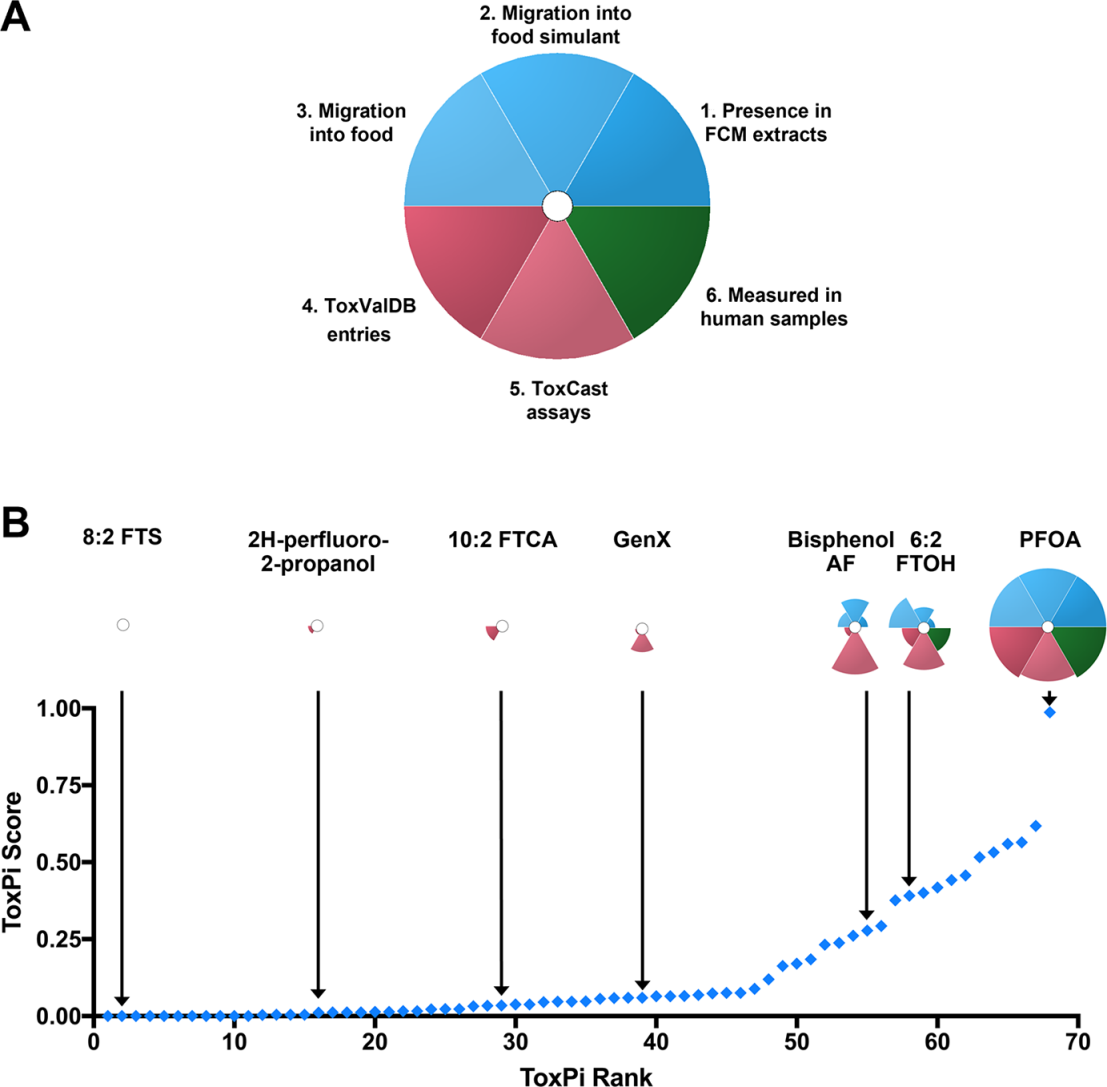
ToxPi analysis highlights hazard data gaps for PFASs in FCCmigex. (A) Overall ToxPi model for ToxPi analysis incorporating data from FCCmigex (1.-3.), USEPA CompTox Dashboard (4.-5.), and human biomonitoring programs and databases (6.). (B) Summary of ToxPi scores for PFASs found in FCCmigex. Compounds are ranked from the highest ToxPi score (1.0) to the lowest ToxPi score (0.0). All individual ToxPis can be found in Figure S1.

Exposure to PFASs has been linked to a wide range of negative health effects in multiple different organ systems, which have been reviewed.^34^ However, hazard data for PFASs have mostly come from studies of PFCAs and PFSAs. Few toxicological studies for hazard identification have been performed on their fluorotelomer-based substitutes.^88−90^ In order to visualize the hazard data that are available for the PFASs detected in FCMs, we utilized the Toxicological Prioritization Index (ToxPi). For our ToxPi model (Figure 2a), we focused on understanding if there were hazard data available rather than whether the compound was a positive hit for any end point. Hazard data were collected from USEPA’s CompTox Dashboard based on either the number of “Hazard” entries (known previously as ToxValDB), which curates publicly available *in vivo* rodent and ecotoxicology studies from several sources, including USEPA, European Chemicals Agency (ECHA), United States Agency Toxic Substances Disease Registry (ATSDR), and United States National Toxicology Program (NTP), or based on the number of ToxCast assays where the compound has been tested. ToxCast is part of the Tox21 program, which includes USEPA, US Food and Drug Administration (USFDA), NTP, and two institutes of the National Institutes of Health; its goal is to develop and utilize use new approach methodologies for high-throughput *in vitro* screening.^91,92^ When analyzing these data for the 68 PFASs detected in FCMs within FCCmigex, we found 29 (or nearly 43%) for which there are neither *in vivo* studies, as determined by ToxValDB, nor *in vitro* studies, as determined by ToxCast assays. This represents a major data gap in the toxicity assessment of PFASs present as FCCs.

Conversely, 39 of these compounds have been tested in *in vivo* and *in vitro* assays. PFOA ranked highest in our ToxPi analysis (ToxPi score of 0.9874 out of 1.0; Figure 2b) because it had the most FCCmigex entries and because copious amounts of hazard data were available. Other PFAAs were ranked behind PFOA in this analysis due to their number of FCCmigex entries, but they also lacked *in vivo* studies compared to PFOA. The first non-PFAA compound that was prioritized in this analysis was 6:2 fluorotelomer alcohol (FTOH), due to its detection in food matrices and testing in *in vitro* assays. 6:2 FTOH was phased into production by manufacturers to avoid the use of potential precursors to long-chain PFCAs.^93^ Its toxicity has been reviewed and compared to that of PFHxA, and previous research deemed that, based on available data, it was more toxic than PFHxA, a short-chain PFCA.^88^ However, the authors note that 5:3 fluorotelomer acid, a bioaccumulative metabolite of 6:2 FTOH with little publicly available *in vivo* hazard data, may be the “predominant driver” of the toxicity.^88^ 6:2 FTOH, once ingested, is metabolized to PFHxA, PFHpA, and 5:3 fluorotelomer acid.^94,95^ Given that fluorotelomer-based compounds can be metabolized to PFCAs and other PFASs after uptake,^96−98^ this presents another route of exposure by which humans are exposed to PFCAs.

Among those 39 PFASs detected in FCMs within the FCCmigex for which hazard data exist, several toxicity end points have been investigated. In *in vivo* mammalian studies, PFCAs and PFSAs were often linked to alterations in liver pathology and/or function,^99−105^ and the same was true for kidneys of exposed animals.^99^ Less common effects observed after exposure to PFCAs and PFSAs reported were links to endocrine disruption, including decreased levels of testosterone and estradiol,^99,106,107^ as well as histopathological effects in the thyroid.^99,108^ Immunotoxicity has been detailed across several studies;^109−116^ this end point has been reported and reviewed in numerous studies^117−119^ but has only been investigated for a small number of PFASs, largely consisting of PFCAs and PFSAs. Uncommonly observed effects of PFCAs and PFSAs that have been addressed in these *in vivo* studies were increased cholesterol,^106,107,120^ reproductive toxicity,^106,121,122^ and neurotoxicity.^123−125^ Such adverse outcomes already identified through existing studies should logically serve as a basis for the risk assessment and management of PFASs in FCMs. For the *in vitro* studies incorporated into our ToxPi model, we focused solely on whether *in vitro* tests had been performed for a compound rather than whether a compound was a positive hit in any of the assays or whether any hits could be translated into *in vivo* toxicity. With this analysis, we reveal that 43 PFASs have not been tested *in vitro*. Many FCM legislations require the assessment of the hazards and levels of all migrating chemicals, which means that individual risk assessments are needed.^18^ However, PFASs without hazard data currently do not comply with these requirements. Despite the fact that high-throughput screening efforts have become commonplace and are the goal of identifying hazardous chemicals under the principles of toxicology in the 21st century,^91,92,126^ such approaches may be impeded by practical hurdles, such as the unavailability of the chemicals for testing. Therefore, another solution for this deadlock is the application of the precautionary principle and restricting PFASs as a group.^127^

As we discuss the hazards of the 68 PFASs detected in FCMs, it must be remembered that exposure through FCMs to these compounds occurs as a mixture and not just to individual compounds. Most of the studies of PFASs found in FCMs identified multiple PFASs in the same FCMs through either targeted^128^ or nontargeted analyses^68^ or tiered approaches.^129^ This severely complicates assessing risk as few studies of PFAS mixtures have been performed to identify the hazards conferred by exposures to mixtures of PFASs.^130^ However, even with the limited data that are available, it has been reported that PFASs within mixtures may act additively and/or synergistically to exert toxic effects, at least in experimental models.^131−133^ PFAS exposure also occurs through routes other than FCMs, including contaminated drinking water and inhalation of contaminated dust, which further complicates assessing the risk of these compounds.

### Caveats of Comparing Concentrations of PFASs in FCMs

When discussing the presence of PFASs in FCMs, concentrations and their trends over time are of interest, as they inform about potential exposure and may help to understand the sources. In the studies included here, wide ranges of concentrations of PFASs have been reported.^128,134−136^ Importantly, the experimental setups and analytical methods differed across the studies in FCCmigex. Therefore, it must be recognized that comparing concentrations among different studies is difficult, even for a single PFAS, as extraction methods, instrumentation, targeted analytes, and tested products may vary within and among laboratories and over time. Because we could not adequately adjust for these variables, an analysis of concentrations, including trends over time, was not performed.

Even more, identifying and quantifying all PFASs in a sample are a major analytical challenge. Total fluorine and extractable organic fluorine analyses are rapid screening tools to assess the fluorine mass balance. For example, Schultes et al.^51^ tested paper and board FCAs using three types of total fluorine analyses, revealing concentrations of total fluorine from ∼300 to ∼4,000 ppm in the material, of which 0–5.5% were measured in extracts. In addition, targeted analysis of 44 different PFASs was performed in the extracts, and 22 different PFASs were detected and quantified. Importantly, the authors assume that a large amount of extractable organic fluorine was not captured by the compound-specific analysis, such as degradation products or unreacted monomers. These data show why investigating just one or a few selected PFASs does not give a holistic picture of the complex mixture that is possibly present in FCMs. Therefore, measuring a combination of total organic fluorine in the material itself and in the extracts, total oxidizable precursor content,^137^ and hydrolysis reaction products^59,138,139^ as well as applying analytical methods based on gas and liquid chromatography and mass spectrometry would support the identification of individual PFASs and may be necessary to understand the extent of unidentified organic fluorine in a sample. Recently, Strynar et al.^140^ laid out guidelines to identify novel PFAS in many different matrices, including food. A combination of all of these methods may not be used routinely to screen the presence and manage the risks of PFASs in FCMs due to the required resources and time, but they form a toolkit that helps to estimate the amount of overlooked PFASs in general.

### Risk Management of PFASs in FCMs

Besides legal initiatives preventing or resuming the use of new or inactive PFASs in FCMs,^141^ regulations on PFASs mainly target exposure through drinking water and set legally enforceable levels.^142,143^ However, there have been recent initiatives to address PFASs in FCMs and to reduce consumer exposure. Most notably, in 2019, Denmark passed a ban on the use of PFASs in paper- and board-based packaging as well as in cellulose-based packaging that went into effect in 2020.^144^ In 2020, the European Food Safety Authority (EFSA) established a recommended tolerable weekly intake (TWI) of the sum of PFOA, PFOS, PFNA, and PFHxS at 4.4 ng/kg of body weight. EFSA arrived at this level by focusing on children as the most vulnerable subpopulation and using decreased vaccine efficacy as the critical end point, but this TWI is thought to be protective of other adverse health outcomes, too.^63^ Despite adopting this TWI, at the time of writing this publication, there is no harmonized regulation of PFASs in food packaging in the EU. In January 2023, five EU member states submitted a proposal to ECHA to restrict all nonessential uses of PFASs, which includes food packaging.^4^ This measure would ban the use of roughly 10,000 PFASs as well as ban the import of products containing them.

Due to concerns over the toxicity of its metabolites, USFDA reached a voluntary agreement with manufacturers to phase out 6:2 FTOH from food packaging in the United States; this phase-out began in 2021 and is expected to be completed by 2024.^95^ In 2022, a committee within the United States Senate passed the Keep Food Containers Safe From PFAS Act with bipartisan support, which would ban PFASs in food packaging at the federal level.^145,146^ However, this bill failed to pass prior to the end of the congressional term. Because of the lack of federal action, several US states have decided to act independently, enacting their own legislation. Since 2018, six states (New York, Washington, California, Colorado, Maryland, and Hawaii) have banned PFASs in paper- or plant-based food packaging,^147−152^ and five other states (Vermont, Connecticut, Maine, Minnesota, Rhode Island, and Oregon) have banned PFASs from all food packaging materials.^153−158^ Depending on the state, enforcement of this legislation has already begun or will begin in the coming years.

Even in the absence of legislation, manufacturers and purchasers are voluntarily phasing out PFASs from their product lines. To track such announcements, the Food Packaging Forum maintains the freely available Brand and Retailer Initiatives Database (BRID; https://www.foodpackagingforum.org/brand-retailer-initiatives). According to this source, since 2013, at least 32 different companies around the globe have committed to addressing PFASs in food packaging at various points in their supply chain. One retailer removed all microwaveable popcorn from shelves until the manufacturers could provide PFAS-free packaging; other actions by this retail chain have been discussed previously.^159^ Multiple major international fast food chains have pledged to remove PFASs from all consumer-facing packaging by 2025.^160−164^ After results showed concentrations of PFASs exceeding 100 ppm in some of their packaging, one company^165^ worked directly with paper mills to make functional changes in the fiber chemistry of the paper that eliminated the need for adding PFAS.^160^ It is unclear exactly what prompted these shifts, although a recent report of public awareness on PFASs and pressure on manufacturers^166^ may offer some insight into these decisions.

Announcements of such voluntary initiatives and commitments are an excellent step forward, but they do not ensure oversight and enforcement for the safety of all products entering the market. Without proper oversight, these announcements may amount to no more than press releases without measurable actions. As the phase out of chemicals and/or products is discussed, there is always a risk of “greenwashing”, the term coined in the 1980s to describe practices that initially appear environmentally conscious but, in reality, are environmentally neutral or even harmful to environmental and human health.^167^ As an example, molded fiber FCAs were introduced and often touted as compostable in order to ease waste in landfills and reduce greenhouse gases. Many of these products, though, contained PFASs to provide water- and oil-resistance,^14,165,168^ and while the containers may break down during composting, the PFASs remain. This is especially problematic if that compost is applied for use in agriculture; similar issues have been identified with the land application of biosolids.^169−171^ The presence of PFASs in these products led the Biodegradable Products Institute to begin rejecting certification of compostable containers that contained organic fluorine.^172^ Without this action from a nongovernmental organization, it raises the question of whether the presence of PFASs in these products would have been addressed. Removing PFASs from FCMs requires broad and enforceable actions from regulators and other stakeholders. Recently, for example, the development of PFAS-free molded fiber with cellulose nanomaterials was published, indicating that alternatives to PFAS in food packaging are possible.^173^

### Life Cycle Considerations of PFASs in FCMs

When the use of PFASs in FCMs and FCAs is managed, the entire life cycle of these compounds must be taken into account. The production of PFASs, even in polymeric forms, often leads to the creation of PFAS byproducts,^174,175^ which may contaminate FCMs/FCAs. For example, some PFASs, such as PFOA, GenX, and ADONA, are used as processing aids to produce fluorinated polymers and may remain in the final FCM. Lubricants used in forming and extrusion processes often contain fluorinated polymers, such as polytetrafluoroethylene and perfluoropolyether. As has been shown for many types of polymers used as FCMs, unreacted monomers, reaction intermediates, and degradation products migrate from polymers, especially after extended use and/or under certain conditions (e.g., high temperature).^12,42,43,176^

In addition to lowering PFAS exposure to consumers, government regulations and voluntary initiatives limiting or banning the use of PFASs in FCMs/FCAs may contribute to positive knock-on effects both up and down the supply chain. One could expect these actions to reduce exposure for people working in fluorochemical manufacturing facilities and/or at facilities where PFAS-containing FCMs are used, made, and disposed of. Additionally, environmental PFAS pollution resulting from the disposal of FCAs including in leachate from landfills,^21,22^ recycling,^23^ and composting/biodegradation^172^ would also be reduced. Landfill leachate, as an example, can lead to contamination of groundwater, surface water, and by extension, drinking water for human populations.^177,178^ Similarly, PFASs can leach from composted materials after application for agriculture.^179,180^ This supports the notion that elimination of PFAS from FCMs can have additional, wide-reaching effects that are protective to human and environmental health.

### Implications for Regulatory Action

In this study, we compiled evidence for 68 PFASs that have been detected in migrates and extracts of FCMs. Only seven of the 68 PFASs were listed in global FCM regulations and industry inventories as intentional starting substances of FCMs, i.e., almost 90% of these PFASs were either reaction or degradation products, impurities, or added during manufacture without having been included in any of the FCM lists of authorized uses included in the FCCdb. Furthermore, little to no toxicity data are available for many of the PFASs detected in FCMs, presenting a large gap in knowledge about their potential hazards (Figures 2b, S1). For some PFASs that were detected in migrates and extracts of FCMs, such as PFOA, BPAF, and 6:2 FTOH, many adverse health outcomes among different organ systems have been observed. Considering that most PFASs in FCMs were detected by targeted analyses that generally focused on a limited number of analytes, this may result in an incomplete identification of the PFASs actually present in FCMs. This evidence is strongly supported by studies addressing the total fluorine content in FCMs and comparing the levels to the sum of individual PFASs.

The frequent detection of PFASs that were not known to be used and further evidence for unidentified fluorinated compounds in FCMs show the difficulties in managing PFASs in FCMs. Additionally, a restriction of single PFASs could lead to regrettable substitutions as numerous different PFASs are on the market that may have similar functions and could be used as alternatives. These considerations question whether effective risk management strategies can be developed if PFASs are regulated individually. Together with the general persistence of PFASs and the known hazard properties of individual PFASs, these results strongly support a class-based restriction, especially in a sensitive area such as FCMs. Ideally, a restriction would ban PFASs on a global scale to prevent the continued production and use in countries that lack legislation or the capacity for compliance monitoring.^181^

Such an approach for managing PFASs is not a novel concept^182^ and is aligned with the proposed concepts of essential use^159^ and property-based regulation,^183^ the restriction proposal recently introduced in the European Union,^4^ as well as the call for simplifying chemicals by reducing use and by abiding by green chemistry principles.^184^ A class-based phase-out of PFASs in food contact materials, including food packaging, would effectively protect public health while enabling the creation of a safe, circular economy.

## Supporting Information Available

Additional methodological details specifying the hazard data sources; list of 47 studies and reports on PFASs in the FCCmigex database (Table S1); list of 68 PFASs present in FCMs (Table S2); individual ToxPi graphs for each of the 68 PFAS (Figure S1); list of individual ToxPi scores (Table S3) (PDF)
List of 140 PFASs in the FCCdb (Table S4) (XLSX)
