# Supplementary material for: Per- and Polyfluoroalkyl Substances in Food Packaging: Migration, Toxicity, and Management Strategies
Source: Environ Sci Technol. 2024 Mar 19;58(13):5670–84. doi: 10.1021/acs.est.3c03702 (PMC10993423; doi:10.1021/acs.est.3c03702)
Supplement: Supplementary file 1 — es3c03702_si_001.pdf [file es3c03702_si_001.pdf]

Supporting Information

Per- and polyfluoroalkyl substances in food packaging: Migration, toxicity, and management strategies

Drake W. Phelps[a], Lindsey V. Parkinson[b], Justin M. Boucher[b], Jane Muncke[b], Birgit Geueke[b,*]

AFFILIATIONS

[a]Independent Consultant, Raleigh, North Carolina, USA

[b]Food Packaging Forum Foundation, Zürich, Switzerland

*Correspondence related to this publication should be addressed to Birgit Geueke, birgit.geueke@fp-forum.org, Staffelstrasse 10, CH-8045 Zurich, Switzerland.

Content

- Methods: Hazard data sources
- Table S1: References
- Table S2: PFASs present in FCMs
- Figure S1: Individual ToxPis
- Table S3: ToxPi scores

Methods

Hazard data sources:

ECHA (2023) C&L Inventory. Available at https://echa.europa.eu/information-on-chemicals/cl-inventory-database

NITE (2023) Chemical Management – GHS General Information. Available at https://www.nite.go.jp/chem/english/ghs/ghs_index.html

ECHA (2023) Candidate List of Substances of Very High Concern for Authorisation. Available at https://echa.europa.eu/candidate-list-table

OEHHA (2023) The Proposition 65 List. Available at https://oehha.ca.gov/proposition-65/proposition-65-list/

Endocrine Disruptor List (2022) Substances Identified as Endocrine Disruptors at EU Level. Available at https://edlists.org/the-ed-lists/list-i-substances-identified-as-endocrine-disruptors-by-the-eu

ECHA (2007) PBT/vPvB Assessments under the Previous EU Chemicals Legislation. Available at https://echa.europa.eu/information-on-chemicals/pbt-vpvb-assessments-under-the-previous-eu-chemicals-legislation

U.S. EPA (2023) Persistent Bioaccumulative Toxic (PBT) Chemicals Covered by the TRI Program. Available at https://www.epa.gov/toxics-release-inventory-tri-program/persistent-bioaccumulative-toxic-pbt-chemicals-covered-tri

U.S. EPA (2016) Priority Chemicals. Available at https://archive.epa.gov/epawaste/hazard/wastemin/web/html/priority.html

ECHA (2023) PBT Assessment List. Available at https://echa.europa.eu/pbt

Stockholm Convention (2022) All POPs Listed in the Stockholm Convention. Available at http://chm.pops.int/TheConvention/ThePOPs/ListingofPOPs/tabid/2509/Default.aspx

ECHA (2023e) List of Substances Proposed as POPs. Available at https://echa.europa.eu/list-of-substances-proposed-as-pops

Arp HPH and Hale, SE (2019) REACH: Improvement of guidance and methods for the identification and assessment of PMT/vPvM substances. Umweltbundesamt 126. Available at https://www.umweltbundesamt.de/publikationen/reach-improvement-of-guidance-methods-for-the

ECHA (2023b) Search for Chemicals. Available at https://echa.europa.eu/search-for-chemicals

Table S1. List of 47 studies and reports on PFASs that have been detected in migrates and extracts of food contact materials and articles (source: FCCmigex).

Begley TH, White K, Honigfort P, Twaroski ML, Neches R and Walker RA (2005) Perfluorochemicals: potential sources of and migration from food packaging. Food additives and contaminants. https://doi.org/https://doi.org/10.1080/02652030500183474.
Begley TH, Hsu W, Noonan G and Diachenko G (2008) Migration of fluorochemical paper additives from food-contact paper into foods and food simulants. Food Additives and Contaminants. Part A. https://doi.org/https://doi.org/10.1080/02652030701513784.
Benotti MJ, Fernandez LA, Peaslee GF, Douglas GS, Uhler AD and Emsbo-Mattingly S (2020) A forensic approach for distinguishing PFAS materials. Environmental Forensics. https://doi.org/https://doi.org/10.1080/15275922.2020.1771631.
Brenes ALM, Curtzwiler G, Dixon P, Harrata K, Talbert J, Vorst K, Monge Brenes AL, Curtzwiler G, Dixon P, Harrata K, Talbert J and Vorst K (2019) PFOA and PFOS levels in microwave paper packaging between 2005 and 2018. Food Additives and Contaminants: Part B. https://doi.org/https://doi.org/10.1080/19393210.2019.1592238.
Cesen M, Lambropoulou D, Laimou-Geraniou M, Kosjek T, Blaznik U, Heath D and Heath E (2016) Determination of Bisphenols and Related Compounds in Honey and Their Migration from Selected Food Contact Materials. Journal of agricultural and food chemistry. https://doi.org/https://doi.org/10.1021/acs.jafc.6b03924.
Chen Y, Lu Z, Huang S, Li G, Hu Y and Zhong Q (2022) Simultaneous enrichment of bisphenols and polyfluoroalkyl substances by cyclodextrin-fluorinated covalent organic frameworks membrane in food packaging samples. Journal of Chromatography A. https://doi.org/https://doi.org/10.1016/j.chroma.2022.462864.
Chiang C-F, Hsieh DP, Hsu H-C, Chang C-C, Ling M-P, Huang L-L, Chen P-W and Chiang H-H (2012) Chinese Cooking with Ionic Seasonings May Enhance Migration of Perfluorooctanic acid from Food Contact Articles. Journal of Food and Drug Analysis. https://doi.org/https://www.scopus.com/record/display.uri?eid=2-s2.0-84875244373&origin=inward&txGid=6cd179570d6adcf720512262bb89d2d4.
Choi H, Bae IA, Choi JC, Park SJ and Kim M (2018) Perfluorinated compounds in food simulants after migration from fluorocarbon resin-coated frying pans, baking utensils, and non-stick baking

papers on the Korean market. Food Additives and Contaminants. Part A. https://doi.org/https://doi.org/10.1080/19393210.2018.1499677.
Costopoulou D, Vassiliadou I, Zafeiraki E and Leondiadis L (2013) Exposure to the persistent perfluorinated compounds PFCs in Greece. Environmental Science. https://doi.org/https://www.semanticscholar.org/paper/EXPOSURE-TO-THE-PERSISTENT-PERFLUORINATED-COMPOUNDS-Costopoulou-Vassiliadou/cb149dcff6107c1717bbebfe2ed8ece0624efaff.
Dolman S and Pelzing M (2011) An optimized method for the determination of perfluorooctanoic acid, perfluorooctane sulfonate and other perfluorochemicals in different matrices using liquid chromatography/ion-trap mass spectrometry. Journal of Chromatography B. https://doi.org/https://doi.org/10.1016/j.jchromb.2011.05.032.
Fengler R, Schlummer M, Gruber L, Fiedler D and Weise N (2011) Migration of fluorinated Telomer alcohols (FTOH) from food contact materials into food at elevated temperatures. Organohalogen Compound. https://doi.org/https://dev.dioxin20xx.org/wp-content/uploads/pdfs/2011/2107.pdf.
Fengler R, Schlummer M, Wolz G, Gruber L and Franz R (2012) Data on migration of poly-and perfluorinated compounds from Food Contact Materials into Food and Food simulants. Conference Proceeding. https://www.researchgate.net/publication/234056037_Data_on_migration_of_poly-_and_perfluorinated_compounds_from_Food_Contact_Materials_into_Food_and_Food_simulants.
Gebbink WA, Ullah S, Sandblom O and Berger U (2013) Polyfluoroalkyl phosphate esters and perfluoroalkyl carboxylic acids in target food samples and packaging-method development and screening. Environmental Science and Pollution Research. https://doi.org/https://doi.org/10.1007/s11356-013-1596-y.
Han S, Song Y, Hu J, Liu R, Chi Y, Kang A, Deng H and Zhu D (2021) Novel computer-assisted separation prediction strategy for online-enrichment-HPLC-FLD in simultaneous monitoring of bisphenols in children's water bottles. Food Chemistry. https://doi.org/https://doi.org/10.1016/j.foodchem.2020.127766.
Kotthoff M, Muller J, Jurling H, Schlummer M and Fiedler D (2015) Perfluoroalkyl and polyfluoroalkyl substances in consumer products. Environmental Science and Pollution Research International. https://doi.org/https://doi.org/10.1007/s11356-015-4202-7.
Lerch M, Nguyen KH and Granby K (2022) Is the use of paper food contact materials treated with per- and polyfluorinated alkyl substances safe for high-temperature applications? - Migration study

in real food and food simulants. Food Chemistry. https://doi.org/https://doi.org/10.1016/j.foodchem.2022.133375.
Li HK, Wu XF, Wu SL, Chen LC, Kou XX, Zeng Y, Li D, Lin QB, Zhong HN, Hao TY, Dong B, Chen S and Zheng JG (2022) Machine learning directed discrimination of virgin and recycled poly (ethylene terephthalate) based on non-targeted analysis of volatile organic compounds. Journal of Hazardous Materials. https://doi.org/https://doi.org/10.1016/j.jhazmat.2022.129116.
Lian L, Jiang X, Guan J, Qiu Z, Wang X and Lou D (2020) Dispersive solid-phase extraction of bisphenols migrated from plastic food packaging materials with cetyltrimethylammonium bromide-intercalated zinc oxide. Journal of Chromatography A. https://doi.org/https://doi.org/10.1016/j.chroma.2019.460666.
Liu X, Guo Z, Krebs KA, Pope RH and Roache NF (2014) Concentrations and trends of perfluorinated chemicals in potential indoor sources from 2007 through 2011 in the US. Chemosphere. https://doi.org/https://doi.org/10.1016/j.chemosphere.2013.10.001.
Martinez-Moral MP and Tena MT (2012) Determination of perfluorocompounds in popcorn packaging by pressurised liquid extraction and ultra-performance liquid chromatography-tandem mass spectrometry. Talanta. https://doi.org/https://doi.org/10.1016/j.talanta.2012.09.007.
Moreta C and Tena MT (2014) Determination of perfluorinated alkyl acids in corn, popcorn and popcorn bags before and after cooking by focused ultrasound solid?liquid extraction, liquid chromatography and quadrupole-time of flight mass spectrometry. Journal of Chromatography A. https://doi.org/https://doi.org/10.1016/j.chroma.2014.06.018.
Poothong S, Boontanon SK and Boontanon N (2012) Determination of perfluorooctane sulfonate and perfluorooctanoic acid in food packaging using liquid chromatography coupled with tandem mass spectrometry. Journal of Hazardous Materials. https://doi.org/https://doi.org/10.1016/j.jhazmat.2011.12.050.
Poothong S, Boontanon SK and Boontanon N (2013) Extraction procedure optimization for perfluorooctane sulfonate and perfluorooctanoic acid in food packaging determination by LC-MS/MS. Journal of Environmental Science and Health. https://doi.org/https://doi.org/10.1080/03601234.2013.795838.
Sanchis Y, Coscolla C and Yusa V (2019) Comprehensive analysis of photoinitiators and primary aromatic amines in food contact materials using liquid chromatography High-Resolution Mass Spectrometry. Talanta. https://doi.org/https://doi.org/10.1016/j.talanta.2018.08.047.

Sapozhnikova Y and Hoh E (2019) Suspect Screening of Chemicals in Food Packaging Plastic Film by Comprehensive Two-Dimensional Gas Chromatography Coupled to Time-of-Flight Mass Spectrometry. Chromatography Online. https://doi.org/https://www.chromatographyonline.com/view/suspect-screening-chemicals-food-packaging-plastic-film-comprehensive-two-dimensional-gas-chromatogr.
Schaider LA, Balan SA, Blum A, Andrews DQ, Strynar MJ, Dickinson ME, Lunderberg DM, Lang JR and Peaslee GF (2017) Fluorinated Compounds in U.S. Fast Food Packaging. Environmental science & technology letters. https://doi.org/https://doi.org/10.1021/acs.estlett.6b00435.
Schlummer M, Sölch C, Meisel T, Still M, Gruber L and Wolz G (2015) Emission of perfluoroalkyl carboxylic acids (PFCA) from heated surfaces made of polytetrafluoroethylene (PTFE) applied in food contact materials and consumer products. Chemosphere. https://doi.org/https://doi.org/10.1016/j.chemosphere.2014.11.036.
Schultes L and et al. (2019) Total Fluorine Measurements in Food Packaging: How Do Current Methods Perform? Environmental science & technology letters. https://doi.org/https://doi.org/10.1021/acs.estlett.8b00700.
Shoeib T, Hassan Y, Rauert C and Harner T (2016) Poly- and perfluoroalkyl substances (PFASs) in indoor dust and food packaging materials in Egypt: Trends in developed and developing countries. Chemosphere. https://doi.org/https://doi.org/10.1016/j.chemosphere.2015.08.066.
Siao P, Tseng SH and Chen CY (2022) Determination of perfluoroalkyl substances in food packaging in Taiwan using ultrasonic extraction and ultra-performance liquid chromatography/tandem mass spectrometry. Journal of Food and Drug Analysis. https://doi.org/https://doi.org/10.38212/2224-6614.3397.
Siddique S, Zhang G, Coleman K and Kubwabo C (2021) Investigation of the migration of bisphenols from baby bottles and sippy cups. Current Research in Food Science. https://doi.org/https://doi.org/10.1016/j.crfs.2021.08.006.
Still M, Schlummer M, Gruber L, Fiedler D and Wolz G (2013) Impact of Industrial Production and Packaging Processes on the Concentration of Per- and Polyfluorinated Compounds in Milk and Dairy Products. Journal of agricultural and food chemistry. https://doi.org/https://doi.org/10.1021/jf4020137.

Surma M, Wiczkowski W, Zielinski H and Cieslik E (2015) Determination of Selected Perfluorinated Acids (PFCAs) and Perfluorinated Sulfonates (PFASs) in Food Contact Materials Using LC-MS/MS. Packaging Technology and Science. https://doi.org/https://doi.org/10.1002/pts.2140.
Timshina A, Aristizabal-Henao JJ, Da Silva BF and Bowden JA (2021) The last straw: Characterization of per- and polyfluoroalkyl substances in commercially-available plant-based drinking straws. Chemosphere. https://doi.org/https://doi.org/10.1016/j.chemosphere.2021.130238.
Tokranov AK, Nishizawa N, Amadei CA, Zenobio JE, Pickard HM, Allen JG, Vecitis CD and Sunderland EM (2019) How Do We Measure Poly- and Perfluoroalkyl Substances (PFASs) at the Surface of Consumer Products? Environmental science & technology letters. https://doi.org/https://doi.org/10.1021/acs.estlett.8b00600.
Toptanci I, Ketenoglu O and Kiralan M (2022) Assessment of the migration of perfluorinated compounds and primary aromatic amines from PTFE-coated non-stick cookware marketed in Turkey. Environmental Science and Pollution Research. https://doi.org/https://doi.org/10.1007/s11356-022-18783-1.
Vapenka L, Vavrous A, Votavova L, Kejlova K, Dobias J and Sosnovcova J (2016) Contaminants in the paper-based food packaging materials used in the Czech Republic. Journal of Food and Nutrition Research. https://doi.org/https://www.vup.sk/en/en/download.php?bulID=1914.
Vavrous A, Vapenka L, Sosnovcova J, Kejlova K, Vrbik K and Jirova D (2016) Method for analysis of 68 organic contaminants in food contact paper using gas and liquid chromatography coupled with tandem mass spectrometry. Food Control. https://doi.org/https://doi.org/10.1016/j.foodcont.2015.07.043.
Begley TH, White K, Honigfort P, Twaroski ML, Neches R and Walker RA (2005) Perfluorochemicals: potential sources of and migration from food packaging. Food additives and contaminants. https://doi.org/https://doi.org/10.1080/02652030500183474.
Begley TH, Hsu W, Noonan G and Diachenko G (2008) Migration of fluorochemical paper additives from food-contact paper into foods and food simulants. Food Additives and Contaminants. Part A. https://doi.org/https://doi.org/10.1080/02652030701513784.
Benotti MJ, Fernandez LA, Peaslee GF, Douglas GS, Uhler AD and Emsbo-Mattingly S (2020) A forensic approach for distinguishing PFAS materials. Environmental Forensics. https://doi.org/https://doi.org/10.1080/15275922.2020.1771631.

Brenes ALM, Curtzwiler G, Dixon P, Harrata K, Talbert J, Vorst K, Monge Brenes AL, Curtzwiler G, Dixon P, Harrata K, Talbert J and Vorst K (2019) PFOA and PFOS levels in microwave paper packaging between 2005 and 2018. Food Additives and Contaminants: Part B. https://doi.org/https://doi.org/10.1080/19393210.2019.1592238.
Cesen M, Lambropoulou D, Laimou-Geraniou M, Kosjek T, Blaznik U, Heath D and Heath E (2016) Determination of Bisphenols and Related Compounds in Honey and Their Migration from Selected Food Contact Materials. Journal of agricultural and food chemistry. https://doi.org/https://doi.org/10.1021/acs.jafc.6b03924.
Chen Y, Lu Z, Huang S, Li G, Hu Y and Zhong Q (2022) Simultaneous enrichment of bisphenols and polyfluoroalkyl substances by cyclodextrin-fluorinated covalent organic frameworks membrane in food packaging samples. Journal of Chromatography A. https://doi.org/https://doi.org/10.1016/j.chroma.2022.462864.
Chiang C-F, Hsieh DP, Hsu H-C, Chang C-C, Ling M-P, Huang L-L, Chen P-W and Chiang H-H (2012) Chinese Cooking with Ionic Seasonings May Enhance Migration of Perfluorooctanic acid from Food Contact Articles. Journal of Food and Drug Analysis. https://doi.org/https://www.scopus.com/record/display.uri?eid=2-s2.0-84875244373&origin=inward&txGid=6cd179570d6adcf720512262bb89d2d4.
Choi H, Bae IA, Choi JC, Park SJ and Kim M (2018) Perfluorinated compounds in food simulants after migration from fluorocarbon resin-coated frying pans, baking utensils, and non-stick baking papers on the Korean market. Food Additives and Contaminants. Part A. https://doi.org/https://doi.org/10.1080/19393210.2018.1499677.
Costopoulou D, Vassiliadou I, Zafeiraki E and Leondiadis L (2013) Exposure to the persistent perfluorinated compounds PFCs in Greece. Environmental Science. https://doi.org/https://www.semanticscholar.org/paper/EXPOSURE-TO-THE-PERSISTENT-PERFLUORINATED-COMPOUNDS-Costopoulou-Vassiliadou/cb149dcff6107c1717bbebfe2ed8ece0624efaff.

Table S2: List of the 68 PFASs identified in the literature as being present in FCMs. Grouping was applied according to Figure 2. The number of fluorinated carbons indicates how many C-atoms carry at least one F-atom. If a PFAS has several side chains, the fluorinated carbons are shown for the each side chain separately.

PFAS group	Name	Abbreviation or Synonym	CAS Number(s)	Fluorinated Carbons
PFCA	Perfluorobutanoic acid;<br>Perfluorobutanoate	PFBA	375-22-4;<br>45048-62-2	3
	Perfluoropentanoic acid	PFPeA	2706-90-3	4
	Perfluorohexanoic acid	PFHxA	307-24-4	5
	Perfluoroheptanoic acid	PFHpA	375-85-9	6
	Perfluorooctanoic acid	PFOA	335-67-1	7
	Perfluorononanoic acid	PFNA	375-95-1	8
	Perfluorodecanoic acid	PFDA	335-76-2	9
	Perfluoroundecanoic acid	PFUnDA	2058-94-8	10
	Perfluorododecanoic acid	PFDoDA	307-55-1	11
	Perfluorotridecanoic acid	PFTrDA	72629-94-8	12
	Perfluorotetradecanoic acid	PFTeDA	376-06-7	13
	Perfluoropentadecanoic acid	PFPeDA	141074-63-7	14
	Perfluorohexadecanoic acid	PFHxDA	67905-19-5	15
	Perfluoroheptadecanoic acid	PFHpDA	57475-95-3	16
	Perfluorooctadecanoic acid	PFOcDA	16517-11-6	17
PFSA	Perfluorobutanesulfonic acid;<br>Potassium perfluorobutanesulfonate;<br>Perfluorobutanesulfonate	PFBS	375-73-5;<br>29420-49-3;<br>45187-15-3	4
	Perfluorohexanesulfonic acid;<br>Perfluorohexanesulfonate	PFHxS	355-46-4;<br>108427-53-8	6
	Perfluoroheptanesulfonic acid	PFHpS	375-92-8	7
	Perfluorooctanesulfonic acid	PFOS	1763-23-1	8
	Perfluorodecanesulfonic acid	PFDS	335-77-3	10

PASF-based	2-(N-(Perfluorobutylsulfonyl)-N-methylamino)ethanol	N-Methylperfluorobutane sulfonamidoethanol	34454-97-2	4
	Perfluorooctanesulfonamide	PFOSA	754-91-6	8
	N-Methylperfluorooctanesulfonamide	NMePFOSA	31506-32-8	8
	2-(N-Ethylperfluorooctanesulfonamido)acetic acid	NEtFOSAA	2991-50-6	8
	2-(N-Methylperfluorooctanesulfonamido)acetic acid	NMeFOSAA	2355-31-9	8
	Ammonium bis(N-ethyl-2-perfluorooctylsulfonaminoethyl)phosphate; Sodium bis[2-(N-ethylperfluorooctane-1-sulfonamido)ethyl] phosphate	Bis(N-ethyl-2-perfluorooctyl-sulfonaminoethyl) phosphate	30381-98-7; 23282-60-2	8 + 8
Fluoro-telomer-based	4:2 Fluorotelomer sulfonate	4:2 FTS	414911-30-1	4
	6:2 Fluorotelomer sulfonic acid; 6:2 Fluorotelomer sulfonate sodium salt	6:2 FTS	27619-97-2; 27619-94-9	6
	8:2 Fluorotelomer sulfonic acid	8:2 FTS	481071-78-7	8
	10:2 Fluorotelomer sulfonic acid	10:2 FTS	120226-60-0	10
	2-(Perfluorohexyl)ethylphosphonic acid	6:2 FT-based phosphinic acid	252237-40-4	6
	2-(Perfluorohexyl)ethanol	6:2 FTOH	647-42-7	6
	2-(Perfluorooctyl)ethanol	8:2 FTOH	678-39-7	8
	2-(Perfluorodecyl)ethanol	10:2 FTOH	865-86-1	10
	2-(Perfluorododecyl)ethanol	12:2 FTOH	39239-77-5	12
	2-(Perfluorotetradecyl)ethanol	14:2 FTOH	60699-51-6	14
	2-(Perfluorohexadecyl)ethanol	16:2 FTOH	65104-67-8	16
	2-(Perfluorooctadecyl)ethanol	18:2 FTOH	65104-65-6	18
	3-(Perfluoropentyl)propanoic acid	5:3 FTCA	914637-49-3	5
	2-(Perfluorohexyl)ethanoic acid	6:2 FTCA	53826-12-3	6
	3-(Perfluoroheptyl)propanoic acid	7:3 FTCA	812-70-4	7
	2-(Perfluorooctyl)ethanoic acid	8:2 FTCA	27854-31-5	8
	2-(Perfluorodecyl)ethanoic acid	10:2 FTCA	53826-13-4	10
	2H-Perfluoro-2-octenoic acid	6:2 FTUCA	70887-88-6	6
	2H-Perfluoro-2-decenoic acid	8:2 FTUCA	70887-84-2	8
	2H-Perfluoro-2-dodecenoate	10:2 FTUCA	70887-94-4	10
	4:2 Fluorotelomer phosphate monoester	4:2 monoPAP	150065-76-2	4

	6:2 Fluorotelomer phosphate monoester	6:2 monoPAP	57678-01-0	6
	8:2 Fluorotelomer phosphate monoester	8:2 monoPAP	57678-03-2	8
	10:2 Fluorotelomer phosphate monoester	10:2 monoPAP	57678-05-4	10
	6:2 Fluorotelomer phosphate diester;<br>Sodium bis[2-(perfluorohexyl)ethyl] phosphate	6:2/6:2 diPAP	57677-95-9;<br>407582-79-0	6 + 6
	6:2/8:2 Fluorotelomer phosphate diester	6:2/8:2 diPAP	943913-15-3	6 + 8
	8:2 Fluorotelomer phosphate diester;<br>Sodium bis(1H,1H,2H,2H-perfluorodecyl)phosphate	8:2/8:2 diPAP	678-41-1;<br>114519-85-6	8 + 8
	8:2/10:2 Fluorotelomer phosphate diester	8:2/10:2 diPAP	1158182-60-5	8 + 10
	10:2 Fluorotelomer phosphate diester	10:2/10:2 diPAP	1895-26-7	10 + 10
	8:2/12:2 Fluorotelomer phosphate diester	8:2/12:2 diPAP	1578186-42-1	8 + 12
	6:2 Fluorotelomer phosphate triester	6:2/6:2/6:2 triPAP	165325-62-2	6 + 6 + 6
	6:2/6:2/10:2 Fluorotelomer phosphate triester	6:2/6:2/10:2 triPAP	1578186-57-8	6 + 6 + 10
	6:2/8:2/8:2 Fluorotelomer phosphate triester	6:2/8:2/8:2 triPAP	1578186-56-7	6 + 8 + 8
	6:2/8:2/10:2 Fluorotelomer phosphate triester	6:2/8:2/10:2 triPAP	1578186-64-7	6 + 8 + 10
PFECAs	Perfluoro-2-methyl-3-oxahexanoic acid	GenX	13252-13-6	5
	Ammonium 4,8-dioxa-3H-perfluorononanoate	ADONA	958445-44-8	6
Other	Bisphenol AF	BPAF	1478-61-1	1 + 1
	2H-Perfluoro-2-propanol	2H-Perfluoro-2-propanol	920-66-1	2
	Perfluorohexyl phosphonic acid	PFHx phosphinic acid	40143-76-8	6
	1,2,3,4,5,5,6,6-Octafluorobicyclo[2.2.2]oct-2-ene	1,2,3,4,5,5,6,6-Octafluoro-<br>bicyclo[2.2.2]oct-2-ene	31462-62-1	6
	Potassium perfluoro-4-ethylcyclohexanesulfonate	N-Decafluoro-4<br>ethylcyclohexanesulfonate	335-24-0	8
	Perfluorooctylphosphinic acid	PFO phosphinic acid	40143-78-0	8

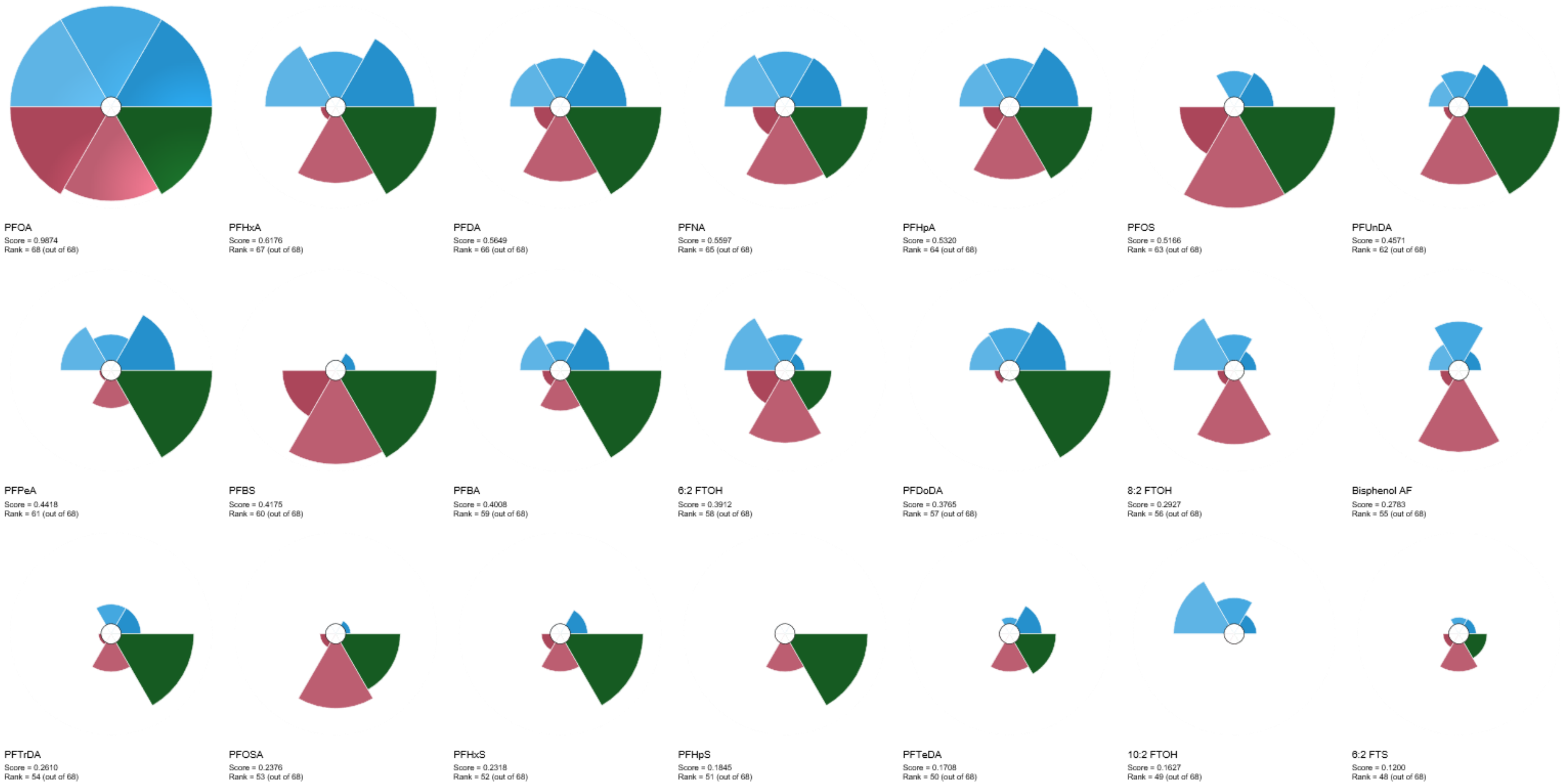

Figure S1: Individual ToxPi graphs for each of the 68 PFAS. Compounds are ranked from highest ToxPi score (1.0) to lowest ToxPi score (0.0).

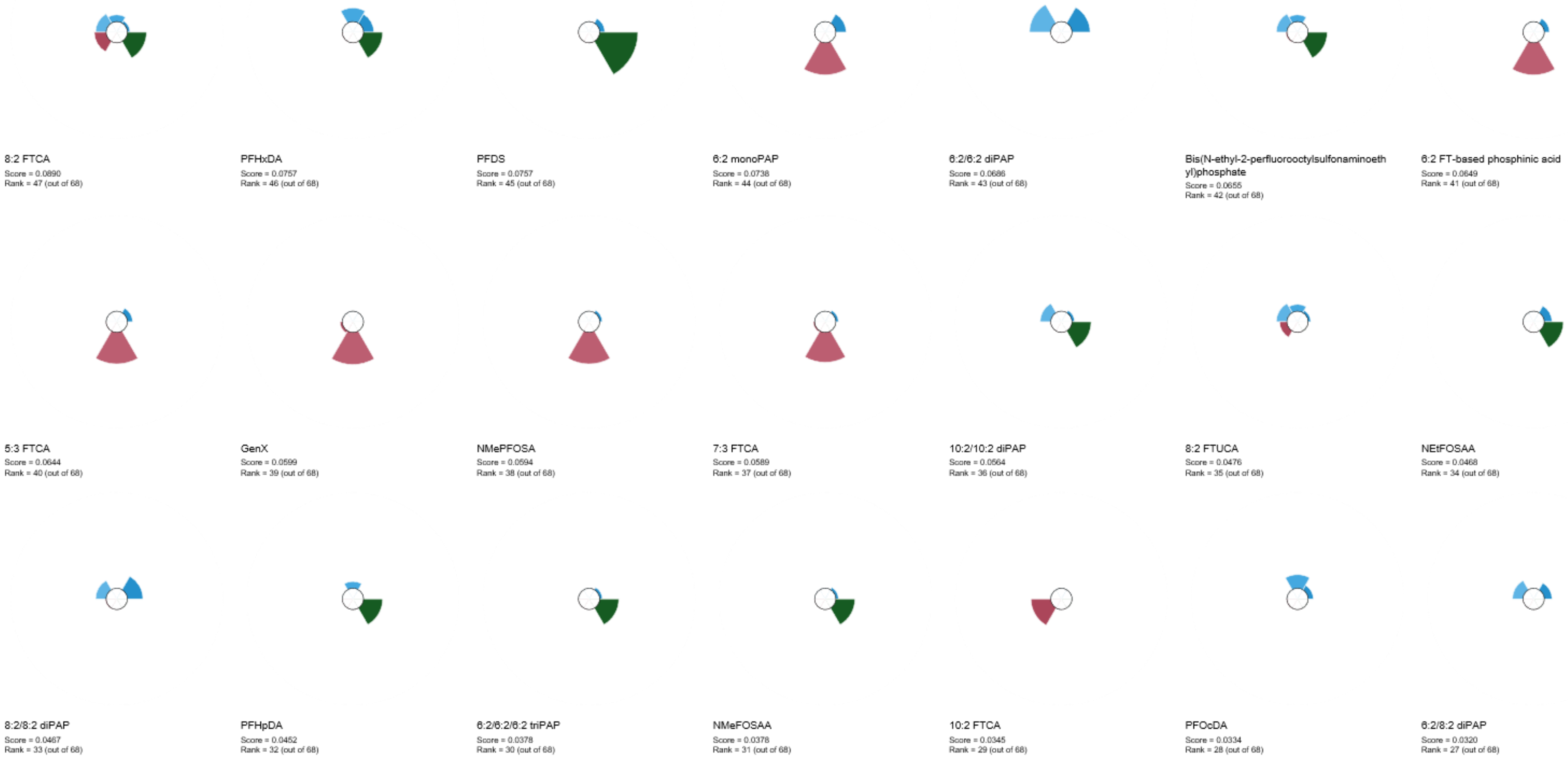

Figure S1, continued

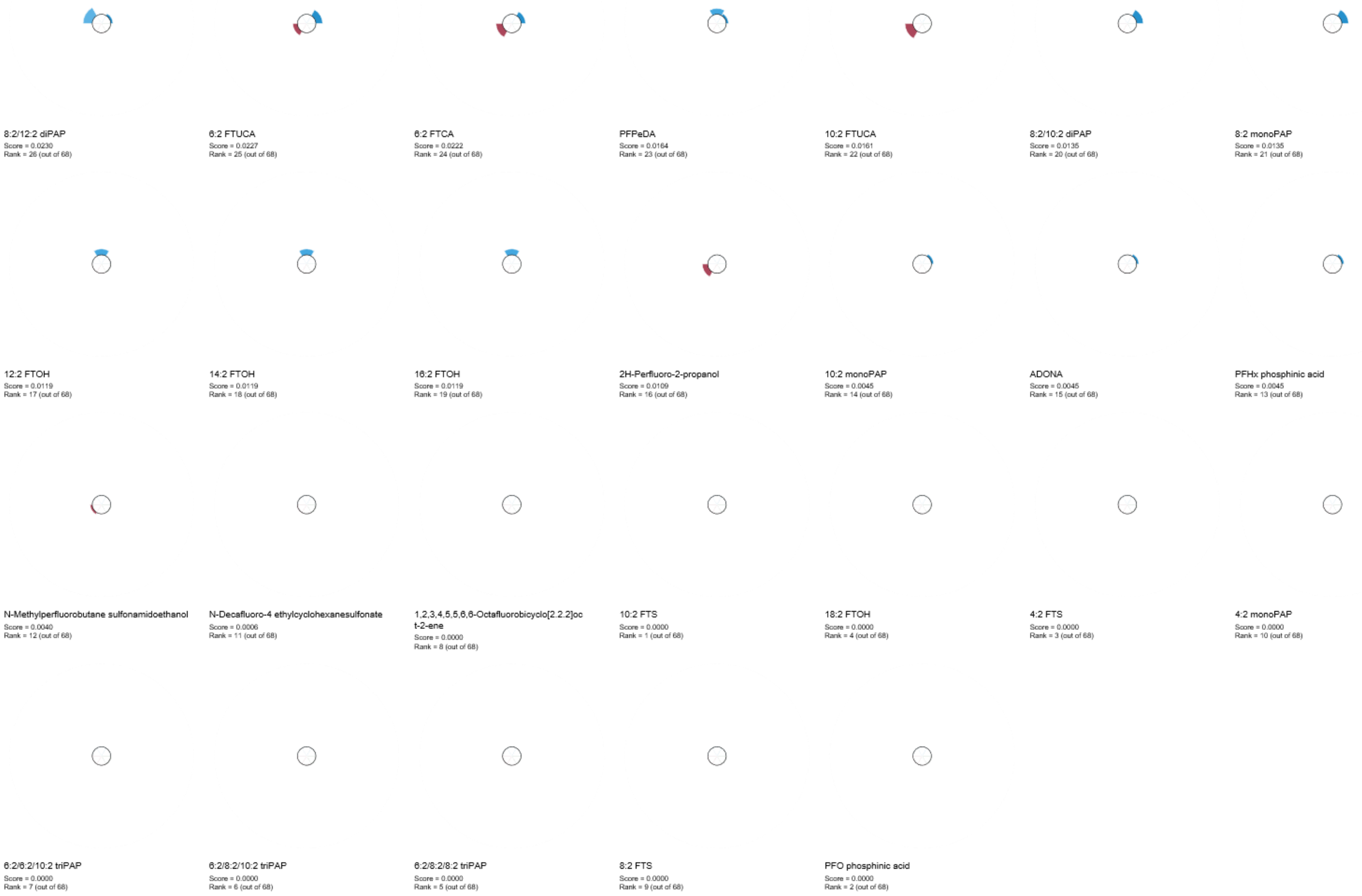

Figure S1, continued

Table S3: ToxPi scores scores for each PFAS ranked from highest to lowest. Individual slice scores are also provided on a scale from 0 to 1. Descriptions of the slices can be found in the Methods.

ToxPi Rank	ToxPi Score	Name	FCCmigex Extraction	FCCmigex Migration into Food Simulant	FCCmigex Migration into Food	ToxValDB Studies	ToxCast Assays Tested	Presence in human biomonitoring studies
68	0.987417	PFOA	1	1	1	1	0.924503	1
67	0.617625	PFHxA	0.756757	0.5	0.666667	0.055172	0.727152	1
66	0.564886	PFDA	0.621622	0.428571	0.444444	0.182759	0.711921	1
65	0.559711	PFNA	0.513514	0.5	0.555556	0.244828	0.744371	0.8
64	0.531973	PFHpA	0.648649	0.428571	0.444444	0.182759	0.687417	0.8
63	0.516616	PFOS	0.324324	0.285714	0	0.489655	1	1
62	0.457116	PFUnDA	0.432432	0.285714	0.222222	0.058621	0.743709	1
61	0.44179	PFPeA	0.594595	0.285714	0.444444	0.02069	0.305298	1
60	0.417527	PFBS	0.108108	0	0	0.475862	0.921192	1
59	0.400801	PFBA	0.432432	0.214286	0.333333	0.089655	0.335099	1
58	0.39119	6:2 FTOH	0.108108	0.285714	0.555556	0.310345	0.687417	0.4
57	0.376527	PFDoDA	0.513514	0.357143	0.333333	0.055172	0	1
56	0.292707	8:2 FTOH	0.135135	0.285714	0.555556	0.075862	0.703974	0
55	0.278316	Bisphenol AF	0.135135	0.428571	0.222222	0.096552	0.787417	0
54	0.261006	PFTrDA	0.216216	0.214286	0	0.027586	0.307947	0.8
53	0.237566	PFOSA	0.054054	0	0	0.062069	0.709272	0.6
52	0.23184	PFHxS	0.189189	0	0	0.096552	0.305298	0.8
51	0.184547	PFHpS	0	0	0	0	0.307285	0.8
50	0.17079	PFTeDA	0.243243	0.071429	0	0.003448	0.306623	0.4
49	0.162734	10:2 FTOH	0.135135	0.285714	0.555556	0	0	0
48	0.119979	6:2 FTS	0.081081	0.071429	0	0.062069	0.305298	0.2
47	0.088951	8:2 FTCA	0.027027	0.071429	0.111111	0.124138	0	0.2
46	0.075736	PFHxDA	0.108108	0.142857	0	0.003448	0	0.2
45	0.075676	PFDS	0.054054	0	0	0	0	0.4

ToxPi Rank	ToxPi Score	Name	FCCmigex Extraction	FCCmigex Migration into Food Simulant	FCCmigex Migration into Food	ToxValDB Studies	ToxCast Assays Tested	Presence in human biomonitoring studies
44	0.073758	6:2 monoPAP	0.108108	0	0	0	0.334437	0
43	0.068569	6:2/6:2 diPAP	0.189189	0	0.222222	0	0	0
42	0.065481	Bis(N-ethyl-2-perfluorooctylsulfonaminoethyl)phosphate	0	0.071429	0.111111	0.010345	0	0.2
41	0.064859	6:2 FT-based phosphinic acid	0.054054	0	0	0	0.335099	0
40	0.06444	5:3 FTCA	0.054054	0	0	0.003448	0.329139	0
39	0.059873	GenX	0	0	0	0.024138	0.335099	0
38	0.059361	NMePFOSA	0.027027	0	0	0	0.329139	0
37	0.0589	7:3 FTCA	0.027027	0	0	0.013793	0.312583	0
36	0.056356	10:2/10:2 diPAP	0.027027	0	0.111111	0	0	0.2
35	0.047571	8:2 FTUCA	0.027027	0.071429	0.111111	0.075862	0	0
34	0.046847	NEtFOSAA	0.081081	0	0	0	0	0.2
33	0.046695	8:2/8:2 diPAP	0.162162	0	0.111111	0.006897	0	0
32	0.045238	PFHpDA	0	0.071429	0	0	0	0.2
31	0.037838	6:2/6:2/6:2 triPAP	0.027027	0	0	0	0	0.2
30	0.037838	NMeFOSAA	0.027027	0	0	0	0	0.2
29	0.034483	10:2 FTCA	0	0	0	0.206897	0	0
28	0.033393	PFOcDA	0.054054	0.142857	0	0.003448	0	0
27	0.032032	6:2/8:2 diPAP	0.081081	0	0.111111	0	0	0
26	0.023023	8:2/12:2 diPAP	0.027027	0	0.111111	0	0	0
25	0.022709	6:2 FTUCA	0.081081	0	0	0.055172	0	0
24	0.022227	6:2 FTCA	0.054054	0	0	0.07931	0	0
23	0.016409	PFPeDA	0.027027	0.071429	0	0	0	0
22	0.016092	10:2 FTUCA	0	0	0	0.096552	0	0
21	0.013514	8:2/10:2 diPAP	0.081081	0	0	0	0	0
20	0.013514	8:2 monoPAP	0.081081	0	0	0	0	0
19	0.011905	12:2 FTOH	0	0.071429	0	0	0	0
18	0.011905	14:2 FTOH	0	0.071429	0	0	0	0

ToxPi Rank	ToxPi Score	Name	FCCmigex Extraction	FCCmigex Migration into Food Simulant	FCCmigex Migration into Food	ToxValDB Studies	ToxCast Assays Tested	Presence in human biomonitoring studies
17	0.011905	16:2 FTOH	0	0.071429	0	0	0	0
16	0.01092	2H-Perfluoro-2-propanol	0	0	0	0.065517	0	0
15	0.004505	10:2 monoPAP	0.027027	0	0	0	0	0
14	0.004505	ADONA	0.027027	0	0	0	0	0
13	0.004505	PFHx phosphinic acid	0.027027	0	0	0	0	0
12	0.004023	N-Methylperfluorobutane sulfonamidoethanol	0	0	0	0.024138	0	0
11	5.75E-04	N-Decafluoro-4 ethylcyclohexanesulfonate	0	0	0	0.003448	0	0
10	0	1,2,3,4,5,5,6,6-Octafluorobicyclo[2.2.2]oct-2-ene	0	0	0	0	0	0
9	0	10:2 FTS	0	0	0	0	0	0
8	0	18:2 FTOH	0	0	0	0	0	0
7	0	4:2 FTS	0	0	0	0	0	0
6	0	4:2 monoPAP	0	0	0	0	0	0
5	0	6:2/6:2/10:2 triPAP	0	0	0	0	0	0
4	0	6:2/8:2/10:2 triPAP	0	0	0	0	0	0
3	0	6:2/8:2/8:2 triPAP	0	0	0	0	0	0
2	0	8:2 FTS	0	0	0	0	0	0
1	0	PFO phosphinic acid	0	0	0	0	0	0
